# Epidemiological, Clinical, and Histopathological Features of the Head and Neck Region Schwannomas—Our Experience in the Western Part of Romania with Surgical Insights

**DOI:** 10.3390/diagnostics14202334

**Published:** 2024-10-20

**Authors:** Adrian Nicoara, Marina Rakitovan, Raluca Maria Closca, Marius Militaru, Alexandru-Cristian Cindrea, Flavia Zara

**Affiliations:** 1 Discipline of Dentoalveolar Surgery, “Victor Babes” University of Medicine and Pharmacy, 300041 Timisoara, Romania; nicoara.adrian@umft.ro; 2 Oro-Maxillo-Facial Surgery Clinic, Emergency City Hospital, 300062 Timisoara, Romania; 3 Department of Microscopic Morphology, University of Medicine and Pharmacy “Victor Babes”, 300041 Timisoara, Romania; raluca.moaca@umft.ro (R.M.C.); flavia.zara@umft.ro (F.Z.); 4 Department of Pathology, Emergency City Hospital, 300254 Timisoara, Romania; 5 Department of Neuroscience, Discipline of Neurology II, “Victor Babes” University of Medicine and Pharmacy, Municipal Emergency Hospital Timisoara, 300041 Timisoara, Romania; marius.militaru@umft.ro; 6 Department of Surgery, “Victor Babes” University of Medicine and Pharmacy, 300041 Timisoara, Romania; alexandru.cindrea.umfvbt@gmail.com; 7 Department of Emergency, Emergency City Hospital, 300079 Timisoara, Romania

**Keywords:** oral pathology, maxillofacial pathology, head and neck schwannomas, immunohistochemical reaction

## Abstract

Introduction: Schwannomas are benign solitary, slow-growing, encapsulated, asymptomatic tumors arising from Schwann’s sheath of nervous fibers. Objectives: The current paper presents all the cases diagnosed with different types of schwannomas in the head and neck region between January 2009 and December 2023 in the Western part of Romania. In this period, ten cases of schwannoma were identified. Materials and Methods: The pathological exams were performed at the Department of Pathology using morphological Hematoxylin–Eosin staining. In addition, immunohistochemical reactions were used in order to confirm the diagnosis. Results: Demographic and clinical data, imaging features, surgical approach, and morphological and immunohistochemical aspects are presented. The results also display an impressive clinical picture of a gigantic ancient schwannoma. Conclusions: This retrospective study describes our experience with head and neck schwannomas, the diagnostic methods available, the surgical decisions, and the histopathological aspects while analyzing the data and reviewing the published specialized literature in the English language.

## 1. Introduction

Schwannoma is an uncommon benign neurogenic tumor originating from the Schwann cells of peripheral nerves [1,2]. It appears as a solitary, slow-growing, encapsulated, asymptomatic tumor that typically arises in association with a nerve trunk and comprises the myelin sheaths surrounding the peripheral nerve [1,2,3]. Peripheral nerve sheath tumors commonly occur in the head and neck region; however, they are rarely found in the oral cavity [4]. Other common sites include the flexor surface of both upper and lower extremities and, less often, the mediastinum and peritoneum [1,5]. The tongue is the most commonly affected intraoral region, followed by the palate, floor of the mouth, buccal mucosa, lips, jaws, and gingiva [1,3,4].

The tumor may occur at any age but is most common in young and middle-aged adults, between the third and sixth decades, without any particular gender predilection [1,3,5]. Schwannomas remain asymptomatic unless they reach an appreciable size. These tumors are usually solitary lesions; however, in some unusual instances, they can be found as multiples and can even occur in association with von Recklinghausen’s neurofibromatosis [2,3]. Schwannomas nearly never suffer malignant transformation [3].

Conservative surgical excision is the treatment of choice, followed by a good postoperative prognosis, with no recurrence, if completely excised [1,3,5].

## 2. Materials and Methods

### 2.1. Patient Selection and Inclusion Criteria

**Figure 1:**
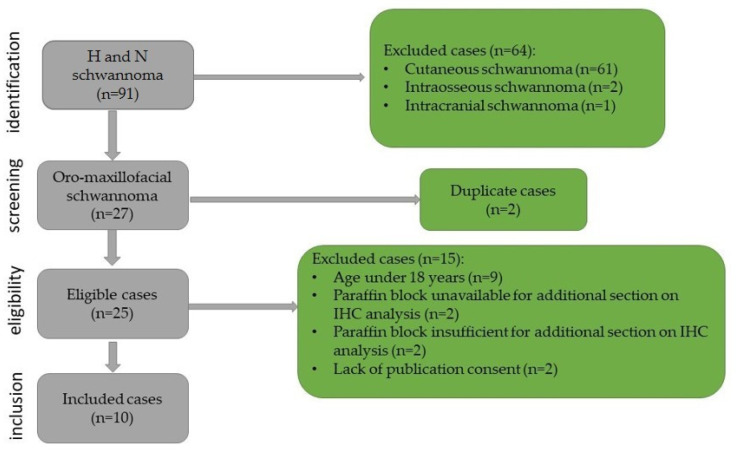
Flowchart for case selection.

This study was retrospective with chronological extension over a period of 14 years, between January 2009 and December 2023. It included all cases of oro-maxillofacial schwannomas diagnosed in the Department of Pathology of the Emergency City Hospital from Timisoara, Romania. The biopsies were performed in the Department of Oro-Maxillo-Facial Surgery and the Ear, Nose, and Throat Clinic. The cases were identified using the registers of the Pathology Department. The inclusion criteria in this study were as follows: patients over the age of 18 years, head and neck tumor site, diagnosis of schwannoma confirmed by histopathological examination, paraffin block available that is sufficient for immunohistochemical staining, and presence of publication consent. The selection process and the exclusion criteria are presented in Figure 1.

### 2.2. Ethical Considerations

In accordance with the ethical norms imposed by the Declaration of Helsinki and in concordance with the Romanian legislation, the opinion of the ethical commission of the hospital was obtained. The annex that accompanies the biological samples that certify the presence of informed patient consent for the use of histologically processed pieces for diagnostic and scientific purposes was checked.

### 2.3. Laboratory Method

The harvested tissue was processed by the standard histological procedure in order to obtain paraffin blocks and perform a histopathological examination. For all cases, three-micrometer-thick serial sections were cut on a Leica RM2245 semi-automated rotary microtome (Leica Biosystems, Nussloch, Germany) and displayed on SuperFrost™ microscope (St. Louis, MO, USA) slides. Hematoxylin–Eosin staining was used for morphological examination.

The histopathological diagnosis was completed using immunohistochemical (IHC) reactions with the following antibodies: anti-S100 protein (S100), anti-Sry-related HMg-Box gene 10 (SOX10), anti-calretinin, anti-cluster of differentiation 56 (CD56), anti-specific neuronal enolase (NSE), anti-glial fibrillary acidic protein (GFAP), anti-vimentin (Vim), anti-cluster of differentiation (CD34), anti-smooth muscle actin (SMA), anti-desmin (Des), anti-podoplanin (D2-40), anti-cluster of differentiation (CD68), and anti-Ki67 proliferation index (Ki67). All the data regarding the antibodies used for IHC reactions are centralized in Table 1.

The panel of antibodies and the reagents utilized for immunohistochemistry were acquired from Novocastra™ (Leica Biosystems, New Castle, UK) and the inclusion time was 30 min. The immunohistochemical staining technique was performed with the Leica Bond-Max automatic device (Leica Biosystems Melbourne Pty Ltd, Australia), following the manufacturer’s standardized protocol.

The histologic and immunohistochemical evaluation was performed by two pathologists with a Leica DM750 microscope. Images were acquired using the Leica DM Share system. 

## 3. Results

### 3.1. Demographic and Clinical Data

**Figure 2:**
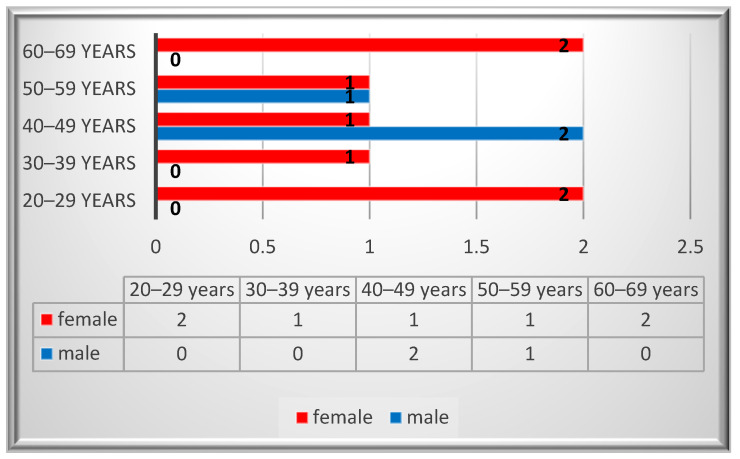
Gender distribution by age.

Ten patients were identified with a diagnosis of schwannoma in the oro-maxillofacial region, with a median age value of 43. The peak incidence was in the 2nd and 6th decades for the female patients, while for the males it was in the 4th decade (Figure 2). A female predominance was noted, with an incidence of 70% (*n* = 7). Most of the patients (80%, *n* = 8) were smokers and two of them reported alcohol consumption. None of the patients reported professional or environmental exposure to toxic agents. 

The patients presented various symptoms, depending on the size and site of the lesion. Non-specific symptoms were noticed, ranging from partial nasal obstruction or discrete discomfort and masticatory issues to facial paresis and dyspnea. Neuropathic pain was not present in the cases presented in this study. The median value of the tumors was 2.25, with half of the cases (*n* = 5) measuring under 2 cm in diameter. The nasal cavity was the most frequently involved tumoral site (*n* = 2). The average time span for hospital presentation was six months (Table 2).

The clinical aspect of the 10th case was quite impressive. The patient presented an important hemifacial asymmetry due to the presence of a well-defined tumefaction involving the left cheek, parotid, and submandibular region. The overlying skin had a reddish color, with areas of a violet tinge. Upon external palpation, a high consistency mass was perceived. The mass was extended superiorly in the temporal region, posteriorly in the subauricular and retromandibular region, and inferiorly in the submandibular territory. At the intraoral examination, a gigantic mass was visible at the level of the jugal mucosa, extended parapharyngeal, crossing the midline, and obstructing the oropharyngeal isthmus. Cranially, the mass reached the lateral upper fornix of the mouth, displacing the contour of the maxillary alveolar ridge medially. The mass had an irregular shape, with well-delimited margins and a smooth surface. The covering mucosa was of a normal aspect, congested in some areas, without the presence of ulcerated or hemorrhagic lesions or discharge of secretions. During the palpation, a firm but elastic consistency was perceived, slightly depressible in the central oropharyngeal area of the mass, fixed to the underlying tissues, without the presence of sensitivity or pain. The movements of the tongue were not impaired.

### 3.2. Imaging Aspects

Due to the small dimensions of the tumors and their localization, imaging investigations were not performed in most of the cases. The vast majority of the tumors were clinically or endoscopically approachable. In one patient, computed tomography was performed due to the large size of the lesion, which was initially elective imaging for this case. The investigation was necessary in order to assess the tumor extension and its neighboring structure involvement.

**Figure 3:**
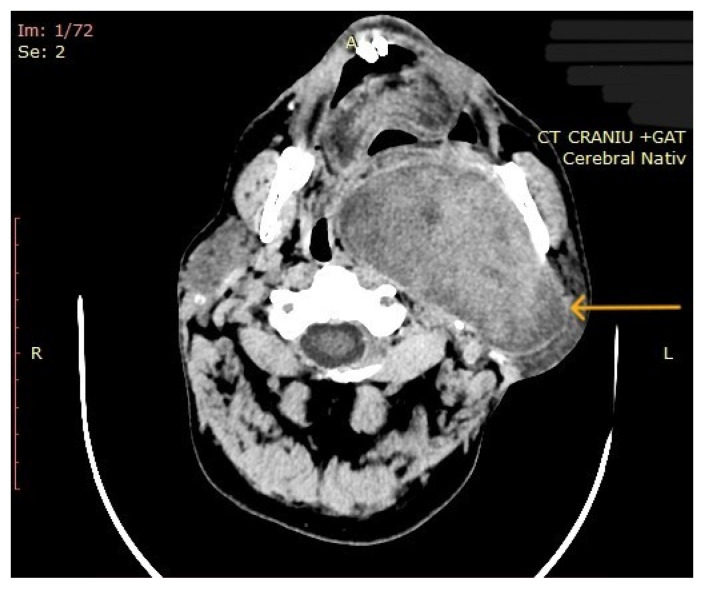
CT imaging: a mass with inhomogeneous uptake of contrast, developed from the left parotid lodge (the arrow represents the tumor mass).

**Figure 4:**
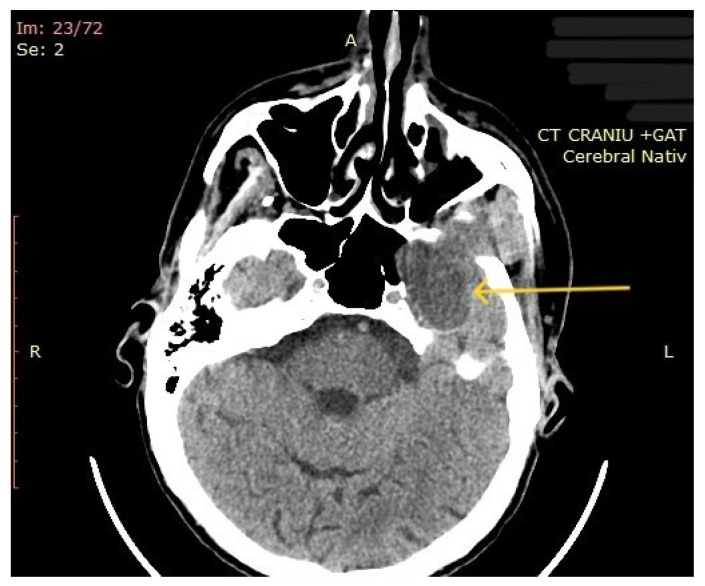
CT imaging: intracerebral component of the mass (the arrow represents the tumor mass).

**Figure 5:**
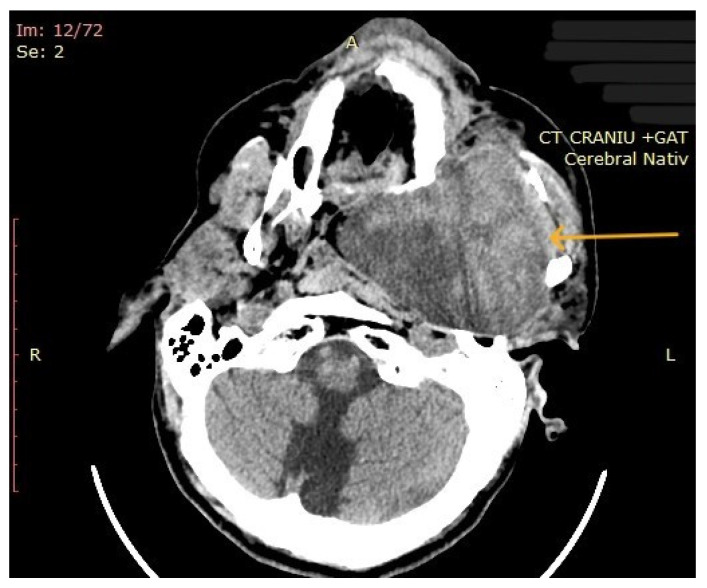
CT imaging: extension of the mass towards the nasopharynx and oro- and hypopharynx (the arrow represents the tumor mass).

The computed tomography (CT) of the head and neck revealed the presence of a large mass measuring 105/76/80 mm, with an inhomogeneous uptake of contrast. The tumor developed from the left parotid lodge (Figure 3) and extended to the left temporal area with osteolysis of the anterior petrous part and the sphenoid bone and thinning of the lateral wall of the left maxillary sinus. The left zygomatic arch was interrupted by a tumoral bud, reaching all the way to the superficial subcutaneous tissue. The intracerebral extension of the mass was 32/29 mm in size (Figure 4). The mass crossed the midline, obstructing the nasopharynx and partially the left half of the oro- and hypopharynx (Figure 5). The carotid and jugular vascular axes on the left side were deformed and displaced posteriorly, as well as the parotid gland, which was displaced laterally.

### 3.3. Involved Nerve and Surgical Approach

**Figure 6:**
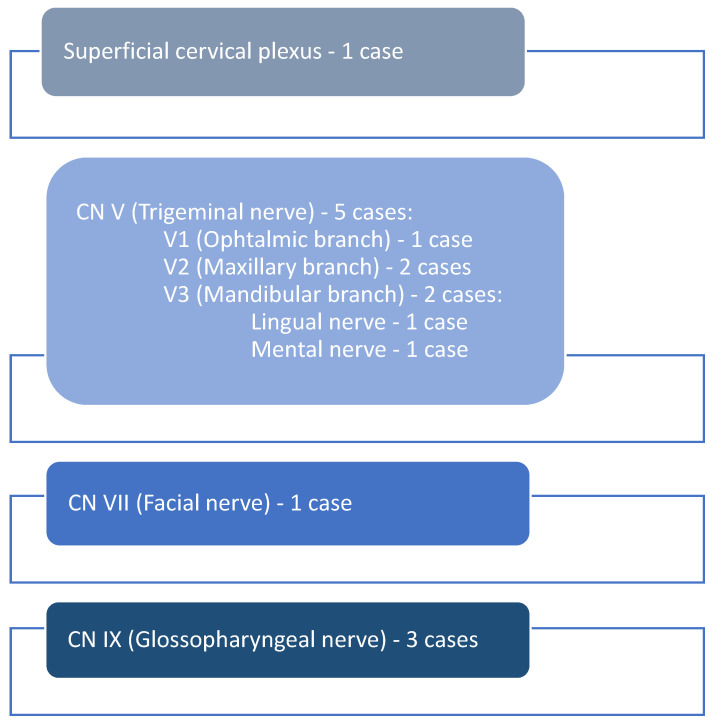
The involved nerves.

Based on the clinical aspects and the tumor site, the originating nerve was identified. The majority of schwannomas (*n* = 5) developed along the branches of the trigeminal nerve, respectively, on the ophthalmic branch (*n* = 1), maxillary branch (*n* = 2), and mandibular branch (*n* = 2), and the lingual (*n* = 1) and mental nerve (*n* = 1). The facial nerve (*n* = 1) and a branch of the superficial cervical plexus (*n* = 1) were involved in one case each, while the glossopharyngeal nerve was involved in three cases (*n* = 3) (Figure 6).

**Figure 7:**
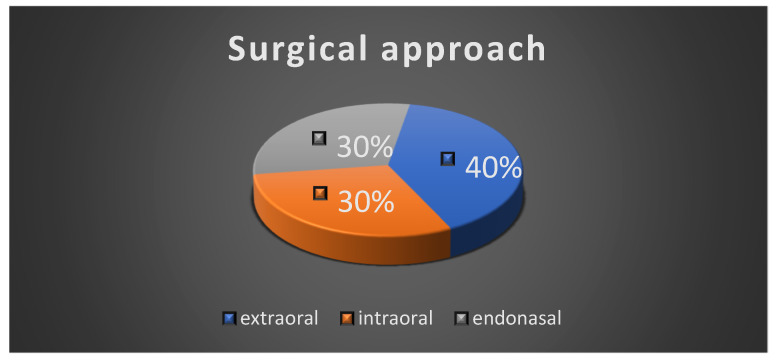
Surgical approach.

All the patients underwent surgical intervention, either complete excision or incisional biopsy of the tumor. The extraoral surgical approach was performed in four cases; three cases had an intraoral surgical approach and three of the patients had an endonasal approach due to the localization of the tumor (Figure 7).

Under local or general anesthesia, a complete surgical excision was performed for eight of the patients. All lesions were easily detached and removed from the underlying structures. The lesions presented no invasiveness in the surrounding tissues and, postoperatively, no residual tumoral tissue remained, macroscopically. Two of the patients underwent incisional biopsy. The nerve of origin was preserved in nine patients (*n* = 9), excepting one case in which the nerve was harvested for biopsy (Table 3). Regarding this last patient, the facial nerve was already involved in the tumor mass and clinically affected, while also being led by the intraoperative aspect of the tumor. The decision to perform radical tumor resection in the block with the interested nerve was made and the technique of enucleation was ruled out.

Subtotal resection was not considered an option by our surgeons for the cases presented in this study, while CyberKnife was not available at all. To the best of our knowledge, CyberKnife is a costly treatment option that is rarely available in Romania.

Because neurostimulators were not available to our clinicians at the specific time period, stimulation and neuromonitoring were not performed, although they are highly recommended in these nervous system tumors. Thankfully, an intraoperative nerve monitoring and stimulation system was available in our clinic during this year, so we use it for operations where there is a risk of nerve involvement. 

### 3.4. Morphological Aspects

**Figure 8:**
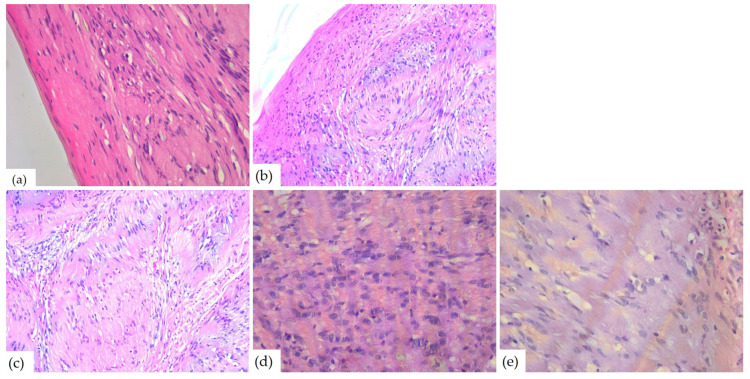
Microscopic aspects of head and neck schwannomas—HE-stained slides: (**a**) tumoral proliferation with thin capsule, ob.40×; (**b**) fusiform cells, ob. 40×; (**c**) Antoni A areas, ob. 40×; (**d**) Antoni B areas, ob. 40×; and (**e**) Verocay body, ob. 20×.

The microscopic examination of Hematoxylin–Eosin-stained slides revealed a well-delimited, encapsulated tumor mass. The tumors had hypercellular Antoni A areas with a fascicular growth pattern, composed of spindle cells, with eosinophilic cytoplasm and elongated, monotonous, blunt-ended nuclei, as well as hypocellular Antoni B areas composed of myxoid stroma (Figure 8).

Due to the microscopic aspects, schwannomas were classified into the following histological subtypes: classic (*n* = 6), cellular (*n* = 2), plexiform (*n* = 1), and ancient (*n* = 1). Half of the cases (*n* = 5) presented stromal and vascular degenerative changes such as vascular or stromal hyalinization, hemorrhage, and hemosiderin deposits (*n* = 4), with cystic degeneration (*n* = 3) and areas of ischemic necrosis (*n* = 1). Three cases showed nuclear changes including nucleomegaly and hyperchromasia and one case also presented increased mitotic activity (Table 4). Most of the cases (*n* = 6) had numerous CD68-positive macrophages in the stroma and three had intracytoplasmic deposition of hemosiderin. Two tumors showed rare mast cells and one had subcapsular lymphocytes.

**Figure 9:**
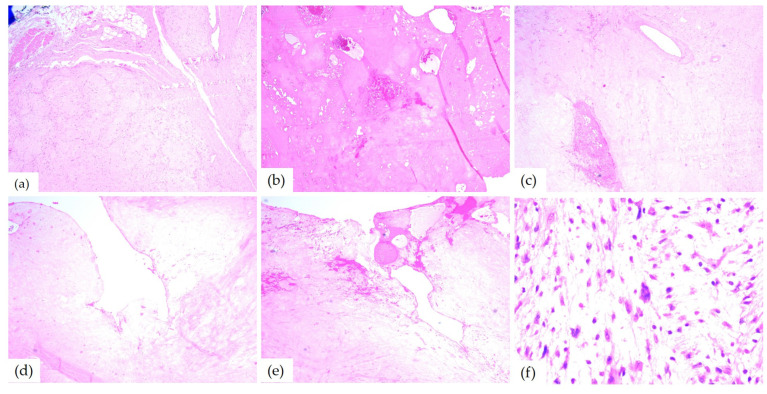
Microscopic aspects of ancient schwannomas—HE-stained slides: (**a**) adjacent to the striated muscle, ob. 4×; (**b**) hyalinization areas and hemorrhages, ob. 4×; (**c**) myxoid areas, ob. 5×; (**d**) cyst formation, ob. 10×; (**e**) cyst formation and hemorrhage, ob. 5×; and (**f**) enlarged hyperchromic and pleomorphic nuclei, ob. 40×.

The microscopic examination of HE-stained slides of the 10th case revealed distinctive aspects of ancient schwannoma: a benign proliferation composed of spindle cells with fine cytoplasmic processes, some with pleomorphic and angular nuclei. Myxoid areas, admixed with foci of hyalinization, cyst formation, as well as hemorrhage were found as well. Between hyalinized collagen bundles, cells with enlarged hyperchromic and pleomorphic nuclei (Figure 9) were also found.

### 3.5. Immunohistochemical Aspects

The immunohistochemical study showed a positive reaction for markers of neuronal origin. Thus, the S100 protein was diffusely positive in all cases, with intense (*n* = 7) or moderate (*n* = 2) immunostaining; one case showed intense/moderate reaction. The SOX 10 marker also showed positivity in all cases, with intense (*n* = 7) or moderate (*n* = 3) immunostaining, but with reaction in only 65–85% of the tumor cells (average value of 80%). Markers of neuroendocrine origin, such as calretinin, CD56, and GFAP showed variable positivity. Among them, CD56 was positive in all cases, with weak (*n* = 4) or moderate (*n* = 6) reactions in 70–90% of tumor cells (average value of 80%). Instead, calretinin had a positive reaction only in two cases, with moderate or weak/moderate immunostaining in 60% and, respectively, 55% of the tumor cells, and the specific neuronal enolase was weakly positive in two cases, with a focal reaction of up to 30% of tumor cells.

**Figure 10:**
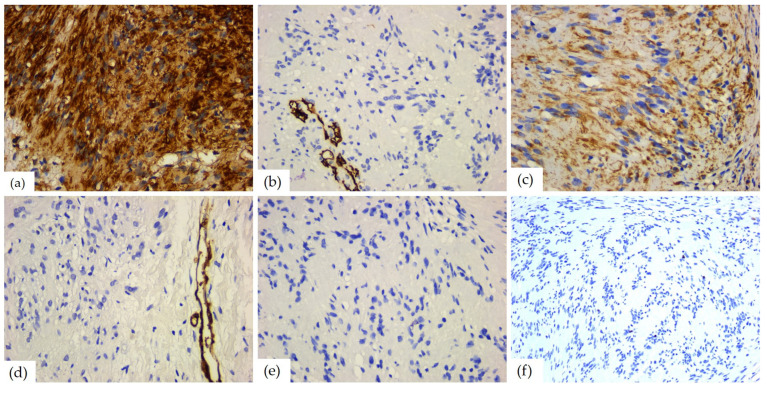
IHC reactions of head and neck schwannomas: (**a**) S100 diffuse and strong positive reaction, ob. 40×; (**b**) CD34 negative reaction, positive internal control present on blood vessels, ob. 40×; (**c**) vimentin, positive reaction in tumor cells, ob. 40×; (**d**) SMA negative reaction, positive internal control present on blood vessels, ob. 40×; (**e**) desmin, negative reaction, ob. 40×; and (**f**) Ki67 mitotic index, ob. 40×.

Glial fibrillary acid protein had a moderate positive reaction in one case, in 5% of the tumor cells. Vimentin, a marker of mesenchymal origin, was positive in all the schwannomas, with weak (*n* = 3), moderate (*n* = 1), or marked (*n* = 6) reaction in 55–100% of the cells (mean value of 90%). Smooth muscle actin, desmin, and CD68 did not show positive immunostaining. Among the markers of vascular origin, podoplanin was negative in all cases, while CD34 was positive in most cases (*n* = 7), with weak (*n* = 1) or moderate (*n* = 6) immunostaining in 5–30% of the tumor cells (average value of 10%). The cell proliferation index was low, with an average value of 2. The aspects of the immunohistochemical study are presented in Table 5 and Figure 10.

### 3.6. Outcome

All of the patients referred to in this article had no postoperative complications due to the surgical procedure. Neurological deficiency was not present in the patients mentioned in this article, with the exception of the patients who had facial paresis prior to hospitalization and surgical intervention.

## 4. Discussion

Schwannoma is a benign neurogenic tumor that is derived exclusively from the Schwann cells of peripheral nerves. Schwann cells play a crucial role in maintaining neural function. They are glial cells that form an important part of the nerve sheath in the peripheral nervous, sensory, and motor systems [1,2,3]. For the first time in history, schwannomas were described by Verocay in 1910 [2,6]. Shortly after, Stout recognized their derivation from Schwann’s cells [3]. In the literature, plenty of alternative terms can be found, with only three of them still in use: the most commonly used are schwannoma, neurinoma, and neurilenoma [1].

Schwannoma is an uncommon benign, solitary, slow-growing, epineurium-encapsulated mass that is painless, asymptomatic, commonly develops in association with a nerve trunk, and comprises the myelin sheaths surrounding the peripheral nerve [1,2,3]. It may occur from cranial and spinal nerve roots or from peripheral nerves, but has a predilection for sensory nerves [1,7,8]. Only 50% of these tumors have a direct relation with a nerve. The most commonly affected nerve is the VIII cranial nerve (acoustic nerve) [3,9].

The benign peripheral nerve sheath tumors (BPNSTs) are mainly represented by schwannoma and neurofibroma. These tumors are the most frequent neurogenic tumors that commonly occur in the head and neck region, representing about 25–45% of all schwannomas, with a small number of cases located elsewhere [1,3,4,5,6,10,11]. They are rarely encountered in the oral cavity [4,6], with only 1% presenting intraoral origin [1,3]. Some authors found that in the head and neck region, schwannomas are more prevalent than neurofibromas, whereas in the oral cavity, it is vice versa [6]. Other common sites include the flexor surfaces of both upper and lower extremities, while less often sites include the mediastinum and peritoneum. It should be emphasized that the intraoral presentation is extremely rare, yet schwannoma can arise both in soft tissue or bone structure [1,2,4].

The tongue is the most commonly affected intraoral site, followed by the palate, floor of the mouth, lips, and the jaws, and furthermore, by buccal mucosa and the gingiva [1,3,4]. Gallo et al. reported 157 cases of schwannoma: 45.2% of the cases involved the tongue and 13.3% encountered the cheek [1,3,12]. Das Gupta et al. reported 136 cases: 60 cases in the neck region, 10 cases in the parotid gland, nine cases in the cheek, eight in the pharynx, respectively, and eight cases in the tongue [1,3,13]. A total of 18 out of 49 cases found by Kun et al. were in the neck area and 11 cases were in the tongue [1,3,14]. In this study, the implicated regions were as follows: inferior and external orbital angle, nasal fossa, and middle meatus with extension to the nasopharynx, retropharyngeal region, parotid lodge, parapharyngeal region with maxillary and intracranial extension, lateral cervical region, lower lip, and the tip of the tongue.

Any part of the tongue may harbor schwannoma [3]. Thus far, approximately 150 cases of schwannoma of the tongue have been previously documented in the literature, predominantly as small series or single case reports. Notably, in a small series, some authors found a difference regarding the precise localization, a higher number of tumors involving the anterior two-thirds of the tongue, while only half of that number involved the posterior one-third [4]. Mevio et al. reported schwannoma in the ventral part of the tongue, while the tip of the tongue remains the least affected part [3,15]. Relating to the cases presented in this article, the localization of the tip of the tongue represents, as mentioned above, the rarest localization of schwannoma in the oral cavity.

A quarter of schwannomas occur in the head and neck region, commonly originating from the facial nerve [16]; they can occur at any level of the facial nerve, from near the brain stem up to the temporal bone [7]. The facial nerve schwannoma can cause many symptoms: tinnitus, otalgia, hearing loss, vertigo, cephalalgia, nerve paresis, and even trigeminal neuralgia [7]. In the case presented in this article, the patient presented unilateral facial paresis. Due to the multitude of functions of the facial nerve (motor—innervates muscles for facial expression; sensory—provides taste sensation; and parasympathetic—supplies many salivary, mucous, and lacrimal glands), the intraoperative preservation of the facial nerve and its function is of big importance. 

Schwannomas originating from the glossopharyngeal nerve are very rare, especially in solitary masses [17]. Patients can present symptoms such as pain, hearing loss, loss of taste, hoarseness, and even compressive symptoms. However, it is usually asymptomatic due to function compensation of the nerve from the contralateral side [17]. The two patients presented in this study reported foreign body sensation, respectively, masticatory discomfort, phonation impairment, sleep disturbance, regional pain, and fatigue.

The clinical aspect of an extraoral schwannoma is painless swelling or a tender unilateral neck mass, with various localization of variable dimensions and firm consistency, with normal overlying skin [16,17]. Our findings confirmed these data as well.

The tumor may arise over a broad age range, but it is prevailing in young and middle-aged adults, between the third and sixth decades [1,3]. For oral schwannoma, it is no different. It is more commonly found between the ages of 30 and 50. Regarding the affirmative age of tumor appearance in the patients presented in this article, the 4th, 5th, and 6th decades of life were prevalent, while one patient reported decade two and one patient decade three as the occurrence date. A big discrepancy was noted in the patient with lingual schwannoma, who described the evolution of the tumor for 10 years, starting at the age of 11. Nevertheless, in all the presented cases, the age when the tumor occurred highlighted the variety found in the literature.

Williams et al. showed that 83% of the head and neck schwannomas were present in males, while Lucas found a greater predilection for females. Other researchers like Hatziotis and Asprides and Enzinger and Weiss showed an equal distribution between both sexes [1,4,18,19,20,21,22]. Several other works have demonstrated that the difference among the genders is minimal; however, various reports show that females are frequently more affected by oral schwannomas [6].

Due to their non-specific clinical presentation and uncommon occurrence, schwannomas are not instantly included in the differential diagnosis, leading to a delayed diagnosis and further treatment [3].

That 10-year delay between the occurrence of the tumor and seeking medical opinion might be explained by the lack of pain or other important impairments, the progressive and slow growth of the lesion, and the capacity of the patient to cope with the presence of the tumor, as well as the expectancy of spontaneous healing. Therefore, the patient with the massive parapharyngeal schwannoma was referred to the hospital when the disturbances and concerns worsened. All of the elements mentioned can lead to challenges in clinical diagnoses, delayed surgical treatment, as well as histopathological diagnoses. 

The schwannomas of the oral soft tissue appear as a smooth-surfaced submucosal swelling, always resembling other, more frequent benign lesions, for instance, fibrous hyperplasia, fibroma, lipoma, leiomyoma, adenoma, mucocele, or other benign tumors of the salivary glands [1,2,3,6]. In the presented cases, the characteristics of the tumors were overlapping with those of the literature.

As already mentioned, oral schwannoma can develop intraosseous as well. Intraosseous schwannomas were found in the posterior region of the mandible, usually viewed as either unilocular or multilocular radiolucencies on radiographic images, which can usually lead to differential clinical and paraclinical diagnosis of cysts and odontogenic tumors [1,22]. The presented cases did not show any intraosseous origin of the tumor, but the patient with the massive parapharyngeal tumor had bone involvement given by the mass effect of the tumor: osteolysis of the anterior petrous part of the left temporal bone and the left lateral part of the sphenoid bone, thinning of the lateral wall of the maxillary sinus, and the interruption of the left zygomatic arch by a tumoral bud.

The majority of the head and neck schwannomas remain asymptomatic unless they reach significant size. Regardless, depending on the site of the lesion and its mass effect, or even nerve involvement, patients may present symptoms such as dysphagia, pain, hoarseness, cranial nerve neuropathies, and Horner syndrome [3,9,23]. Other symptoms that can appear are dysgeusia and loss of motor and/or sensory function. Uncommonly, patients can suffer from dysarthria or airway compromise, which can appear in some tumors that involve the base of the tongue [4]. Two of the cases described in this article did not present any major symptoms other than a discrete discomfort in the act of mastication and/or phonation, others reported foreign body sensation and unilateral nasal obstruction followed by oral breathing and dyspnea. Two patients acknowledged physiognomic disturbance, one reported unilateral facial paresis, while the last case presented alarming inconveniences, including pain, dysphagia, dysphonia, and dyspnea, influencing the general condition of the patient, including lethargy, minor weight loss, and most likely, the explicable heart condition (sinus tachycardia).

These tumors are usually solitary lesions; however, in some unusual instances, they can be found as multiples and can even occur in association with schwannomatosis and von Recklinghausen’s neurofibromatosis, which is one of the most common inherited disorders in humans, occurring in one of every 2000 to 4000 births [2,3,6,24,25]. In this disease, the tumors clinically present themselves at puberty, maintaining their progressive growth throughout their entire life or until surgical removal [6]. Therefore, the presence of schwannoma demands further exploration for nerve tumors in other sites, although, in most cases, there are none to be found. The differential diagnosis with neurofibroma is essential since a solitary neurofibroma may be a manifestation of neurofibromatosis [1,22].

Because schwannomas share clinical features and imagistic aspects with other tumors and infectious conditions that can appear in the same anatomical area, the gold standard for the diagnosis of schwannomas remains the histopathological exam [26,27,28,29]. In patients with large tumors, magnetic resonance imaging is necessary in order to differentiate schwannomas from other entities such as pleomorphic adenoma, Madelung’s disease, branchial cysts, or vascular malformation. Moreover, intraoperative frozen section examination could help the preservation of the nerve bundles involved in the tumor [16,30,31].

In contrast to multiple neurofibromatosis, schwannomas almost never suffer malignant transformation; however, this topic has been at least controversial [1,3]. Some authors reported malignant schwannomas that represent 5% of all soft tissue sarcomas, with only 9–14% located in the head and neck [3], while others found a malignant transformation rate of 8–13.9% [1]. Approximately 8–10% of schwannomas reported in the head and neck region were malignant, while intraorally the risk of the appearance of malignancy features remains extremely low [2].

Conservative surgical excision is the treatment of choice for oral schwannomas, which is frequently carried out by the intraoral approach. The extraoral approach is usually avoided to prevent visible scarring; however, it can be recommended in some cases depending on the size and the location of the mass. If the nerve of origin is visualized and identified, an attempt should be made to isolate it carefully, in order to preserve its function, although this is sometimes not possible [1,2]. The surgical treatment has a good postoperative prognosis, with no recurrence if completely excised through biopsy or local excision [1,2,3,4,32,33]. For eight of the presented patients with smaller schwannomas, the complete resection of the tumor mass was performed. A good postoperative outcome, without relapse at the three-, six-, and 12-month follow-up was identified. For the tenth case, after obtaining the diagnosis of the biopsied schwannoma, the patient rejected the treatment plan for the removal of the tumor under the interdisciplinary collaboration of radiologists to continue the investigation with magnetic resonance imaging, anesthesiologists, oral and maxillofacial surgeons, otorhinolaryngologists, neurosurgeons, and pathologists. He presented himself for clinical monitoring in the first month and did not return after that.

Microscopically, schwannoma is usually an encapsulated tumor that presents a heterogeneous appearance. The tumor cells had two different growth patterns: Antoni A areas, which consisted of spindle-shaped cells with elongated monotonous nuclei and indistinct cytoplasmic borders arranged in palisade or parallel rows, intermingled with eosinophilic hypocellular areas named Verocay bodies, and Antoni B areas, which consisted of fusiform cells distributed in a loose connective tissue matrix. Also, cystic degeneration and inflammatory cells may be found [6].

Most of the papers reviewed for this article described, from the microscopic point of view, similar aspects. The tumor was encapsulated and well demarcated by a thin fibrous capsule composed of lobules of spindle-shaped cells, which are feature characteristics of schwannomas. Also characteristic are the two patterns of cellular arrangements: Antoni type A and type B. The Antoni A areas present fusiform cells with palisading nuclei forming prominent Verocay bodies that contain free bands of amorphous substance between the rows of nuclei and thin cytoplasmic processes with small amounts of collagen. Antoni B areas are represented by the predominant microscopic pattern that contains a smaller number of cells and less organization, with some Antoni A areas separated by loss of connective tissue [1,3]. The cases from this study presented histopathological aspects similar to the ones found in the literature for classical schwannoma.

According to the literature review, immunohistochemical staining is essential in the diagnosis of this tumor [1]. Specific IHC markers commonly used are for S100 protein and vimentin. Their positivity supports the Schwann cell origin of these tumors [3]. As in the cases presented in this article, all of the tumoral cells in schwannoma are positive for the markers cited [1]. The tumor grows slowly; therefore, the Ki67 cell proliferation index is always low, being 5–8% in the cases presented.

An important finding by Ackerman and Taylor in 1951 stated that some schwannomas went through histological degenerative changes and attributed this phenomenon to the “ageing” of the tumor, naming those tumors ancient schwannomas (AS) [2,34]. These degenerative histopathological changes include hemorrhage with hemosiderin deposits, cyst formation, calcification, hyalinization, and myxoid degeneration [2,35]. Despite these degenerative changes, ancient schwannomas behave similarly to schwannomas [36]. Those atypical changes are due to degeneration and should not be misinterpreted as malignancy [36]. Particular aspects of these tumors are the presence of large hyperchromatic atypical nuclei, often lobulated, but with no mitotic figures [37,38].

The first case of intraoral ancient schwannoma was reported by Eversole and Howell in 1971 [39,40], with research continuing throughout the years by authors worldwide; intraoral ancient schwannoma is still one of the most infrequent types of schwannomas in the oral cavity. In terms of tumor size, oral ancient schwannomas are normally larger compared to conventional schwannomas, which are 10–40 mm [39]. The tenth case described in this article presented histopathological elements of an ancient schwannoma, as well as a massive clinical dimension, making the findings of this article more interesting.

The histopathological differential diagnosis of the schwannoma is made with the neurofibroma, both containing fusiform cells with irregular nuclei arranged between collagen fibers. Cytogenetically and histologically, the schwannoma is derived from the Schwann cells, while the neurofibroma is derived from the fibroblasts of the perineurium. Neurofibroma is an unencapsulated tumor consisting of a mixture of Schwann, perineurial cells, and fibroblasts [1,22]. The schwannomas with predominant Antoni A areas should be differentiated by other benign tumors containing spindle cells, as leiomyoma, myofibroma, or other tumors of myofibroblastic origin, solitary fibrous tumor, hemangioma, and also with malignant tumors with fusiform cells as spindle cell squamous carcinomas, sarcomatoid carcinomas, leiomyosarcoma or achromic melanoma [28,41]. The other immunohistochemical markers performed in this study, CD34, vimentin, desmin, and SMA, were used to differentiate between the tumors composed of spindle cells [6].

Besides other benign tumors, schwannomas should be differentiated from malignant transformation as well. As opposed to benign tumors, malignant schwannomas present a higher mitotic rate with necrotic areas and infiltrative edges. There is an inconstant immunohistochemical positivity for the S100 protein and the Ki67 mitotic index is very high [1,24]. Since it usually develops in the extremities, the malignant variant is very rare in the intraoral site, although Hamakawa et al. described a case in the mandible, with metastatic phenomena in the parotid gland and lung area [1,42]. Kun et al. described six cases in the maxillofacial region, from which two have had malignant transformation [1,14].

Considering the rarity of head and neck schwannomas, the specialized literature offers mostly small case series or single case reports. Comparative data previously mentioned in the discussion of this research can be found in Table 6.

## 5. Conclusions

This paper is a retrospective study of all the schwannomas diagnosed in the head and neck region. Due to its unspecific clinical appearance and, nonetheless, rarity in the head and neck area, establishing a diagnosis can be a challenging task. The final diagnosis relies on pathological examination with specific IHC reactions. The treatment of choice is complete surgical excision, followed by periodical clinical follow-up. This study describes our experience with head and neck schwannomas, the diagnostic methods available, the surgical decisions, and the histopathological aspects while analyzing the data and reviewing the published specialized literature in the English language. It also presents an impressive clinical picture of a gigantic ancient schwannoma with its particularities. Only by reporting all diagnosed cases of schwannomas, as well as studying other already published ones, can this infrequent tumor be brought to light and included in the differential diagnosis of oral and maxillofacial tumoral pathology.

## Figures and Tables

**Table 1:** Data related to the antibodies used for immunohistochemical reactions.

Antibody	Substrate	Clone	Dilution	Antigen Retrieval	Exposure Time
S100	Polyclonal Rabbit	EP32	1:100	^1^ ERS2	20 min
SOX 10	Monoclonal Rabbit	SP267	1:100	^2^ ERS1	10 min
Calretinin	Monoclonal Mouse	CAL6	1:200	ERS2	20 min
CD56	Monoclonal Mouse	CD564	1:300	ERS2	20 min
NSE	Monoclonal Mouse	22C9	^3^ RTU	ERS2	20 min
GFAP	Monoclonal Mouse	GA5	25:1000	ERS1	20 min
Vimentin	Monoclonal Mouse	V9	1:800	ERS1	20 min
CD34	Monoclonal Mouse	QBEnd/10	RTU	ERS2	20 min
SMA	Monoclonal Mouse	asm-1	1:50	ERS1	30 min
Desmin	Monoclonal Mouse	DE-R-11	1:75	ERS2	20 min
D2-40	Monoclonal Mouse	322M-14	1:20	ERS2	30 min
CD68	Monoclonal Mouse	514H12	1:100	ERS2	20 min
Ki67	Monoclonal Mouse	MM1	1:200	ERS2	20 min

^1^ ESR2: Epitope Retrieval Solution 2, pH = 9; ^2^ ESR1: Epitope Retrieval Solution 1, pH = 6; and ^3^ RTU: Ready-to-Use.

**Table 2:** The main clinical aspects of the oro-maxillofacial schwannomas.

Case	Site	Signs and Symptoms	Onset of Symptoms	Size	Presumptive Diagnosis
1	Left parotid lodge	Left facial paresis	6 months	3.5 cm	Pleomorphic adenoma
2	Right retropharyngeal region	Foreign body sensation	3 months	2.5 cm	Benign tumor of soft tissue
3	Left nasal fossa	Dyspnea	1 month	1 cm	Neurofibroma
4	Left middle meatus with extension to the posterior wall of the nasopharynx	Left nasal obstruction, oral breathing	6 months	5 cm	Inverted papilloma/ malignant tumor
5	Inferior and external angle of the left orbit	Physiognomic disturbance	2 years	2 cm	Pleomorphic adenoma
6	Anterior third of the left nasal fossa	Painless	1 month	0.8 cm	Pyogenic granuloma
7	Right latero-cervical region	Painless	6 months	3.5 cm	Lymphadenopathy
8	Tip of the tongue	Slight discomfort when talking, eating, and chewing	10 years	1.5 cm	Traumatic fibroma
9	Central core of the lower lip	Discomfort in mastication	9 months	2 cm	Oral irritation fibroma
10	Left parapharyngeal region with maxillary and intracranial extension	Discomfort in breathing, chewing, swallowing, phonation, sleeping, regional pain, fatigue	5 years	10 cm	Benign tumor of soft tissue

**Table 3:** Surgical technique and approach.

Case	Surgical Procedure	Surgical Approach	Preservation of the Nerve of Origin
1	Parotidectomy with the complete excision of the tumoral mass	Extraoral	No, harvested fragment of the nerve for biopsy
2	Complete excision	Intraoral	Yes
3	Complete excision	Endonasal	Yes
4	Incisional biopsy	Endonasal	Yes
5	Complete excision	Extraoral	Yes
6	Complete excision with underlying cartilage	Endonasal	Yes
7	Complete excision	Extraoral	Yes
8	Complete excision	Intraoral	Yes
9	Complete excision	Intraoral	Yes
10	Incisional biopsy	Extraoral	Yes

**Table 4:** Morphological aspects of head and neck schwannomas.

Case	Histological Subtype	Architectural Pattern	Degenerative Changes	Nuclear Changes	Inflammatory Component
1	Classic	Both Antoni A and Antoni B areas, Verocay bodies, pseudoglandular areas	Vascular and stromal hyalinization, hemorrhage	No nuclear changes	Numerous macrophages and siderophages
2	Classic	Predominantly the hypocellular Antoni B areas	Cystic degeneration, ischemic necrosis	No nuclear changes	Numerous mast cells and rare macrophages
3	Classic	Both Antoni A and Antoni B areas, Verocay bodies	No degenerative changes	No nuclear changes	Very rare macrophages
4	Cellular	Predominantly the hypercellular Antoni A areas, devoid of Verocay bodies	Rare vascular vessels with hyalinization	Nucleomegaly and hyperchromasia	Numerous macrophages
5	Cellular	Predominantly the hypercellular Antoni A areas, devoid of Verocay bodies	No degenerative changes	Nucleomegaly and hyperchromasia	Subcapsular lymphocyte
6	Classic	Both Antoni A and Antoni B areas, Verocay bodies	No degenerative changes	Sporadic hyperchromic nuclei	Very rare macrophages
7	Classic	Both Antoni A and Antoni B areas, Verocay bodies	Vascular hyalinization, hemorrhage, hemosiderin deposits, cystic degeneration	No nuclear changes	Numerous macrophages and siderophages, rare mast cells
8	Plexiform	Plexiform pattern with Antoni A and Antoni B areas	No degenerative changes	No nuclear changes	Numerous macrophages
9	Classic	Both Antoni A and Antoni B areas, Verocay bodies	No degenerative changes	Sporadic hyperchromic nuclei	Numerous macrophages, rare mast cells
10	Ancient	Both Antoni A and Antoni B areas, Verocay bodies	Extensive stromal hyalinization, hemorrhage, hemosiderin deposits, cystic degeneration	Enlarged hyperchromic and pleomorphic nuclei, mitoses	Numerous macrophages and siderophages

**Table 5:** Immunohistochemical data.

IHC Marker	Case 1	Case 2	Case 3	Case 4	Case 5	Case 6	Case 7	Case 8	Case 9	Case 10
S100	^1^ +++ Diffuse	^2^ ++ Diffuse	+++ Diffuse	+++ Diffuse	+++ Diffuse	+++ Diffuse	^3^ +++/++ Diffuse	+++ Diffuse	+++ Diffuse	++ Diffuse
SOX10	^4^ ++/+++ 80%	++/+++ 65%	+++ 80%	++ 80%	+++ 80%	+++ 70%	+++ 85%	+++ 75%	+++ 85%	+++ 80%
Cal	^5^ -	-	-	-	++ 60%	^6^ +/++ 55%	-	-	-	-
CD56	^7^ + 85%	+/++ 80%	+/++ 70%	+ 80%	^8^ ++/+ 80%	++ 75%	++ 90%	++ 80%	+/++ 90%	++/+ 70%
NSE	+ Focal	+ 30%	-	-	-	-	-	+ 10%	-	-
GFAP	++ 5% of cells	-	-	-	-	-	-	-	-	-
Vim	+++ 80%	+++ 80%	+++ 90%	+++ 90%	+/++ Diffuse	+++ 90%	+ Diffuse	++ Diffuse	+++ 95%	+/++ 55%
SMA	− ^9^ PIC+	− PIC+	− PIC+	− PIC+	− PIC+	− PIC+	− PIC+	− PIC+	− PIC+	− PIC+
Des	-	-	-	-	-	-	-	-	-	-
CD34	+ 10%	++ 30%	++ 15%	− PIC+	− PIC+	++ 5%	++ 5%	− PIC+	++ 5%	++ 15%
D2-40	-	-	-	-	-	-	-	-	-	-
CD68	− PIC+	− PIC+	− PIC+	− PIC+	− PIC+	− PIC+	− PIC+	− PIC+	− PIC+	− PIC+
Ki67	6%	1%	1%	6%	3%	2%	1%	2%	2%	5%

^1^ +++: intense positive reaction; ^2^ ++: moderate positive reaction; ^3^ +++/++: intense/moderate reaction; ^4^ ++/+++: moderate/intense reaction; ^5^ −: negitve reaction; ^6^ +/++: weak/moderate reaction; ^7^ +: weak positive reaction; ^8^ ++/+: moderate/weak reaction; ^9^ PIC: positive internal control.

**Table 6:** Case series of schwannoma published in the literature and reviewed in our study.

Authors	Year of Publication	Journal Abbreviation	Type of Study	Number of Schwannoma Cases
Lambade et al. [1]	2015	*J. Oral Maxillofac. Surg.*	^1^ N/A	1
Nassehi et al. [2]	2021	*BMJ Case Rep.*	Review	1
Thompson et al. [4]	2020	*Head Neck Pathol*	N/A	19
López-Carriches et al. [5]	2009	*Med Oral Patol Oral Cir Buccal*	Review	1
do Nascimento et al. [6]	2011	*Clin Oral Investig*	N/A	4
Gallo et al. [12]	1977	*J Am Dent Assoc*	Case Report	5
Mevio et al. [15]	2022	*Rev Laryngol Otol Rhinol (Bord)*	Case Report	1
Tawfeeq et al. [17]	2023	*Int. J. Surg. Case Rep.*	Case Report	1
Martins et al. [22]	2009	*Indian J Dent Res*	Review	1
Lira et al. [23]	2013	*Acta Otorhinolaryngol Ital*	N/A	1
Baderca et al. [26]	2008	*Rom J Morphol Embryol*	Review	1
Kim et al. [38]	2011	*J Korean Assoc Oral Maxillofac Surg*	N/A	2

^1^ N/A: not available.

## Data Availability

The data that support the fundings on this study are available from the corresponding author upon reasonable request.
